# Supplementary material for: Comparison of diagnostic accuracy of 2D and 3D measurements to determine opportunistic screening of osteoporosis using the proximal femur on abdomen-pelvic CT
Source: PLoS One. 2022 Jan 4;17(1):e0262025. doi: 10.1371/journal.pone.0262025 (PMC8726491; doi:10.1371/journal.pone.0262025)
Supplement: Attachment — Submitted filename: Reponse to reviewers.docx [file pone.0262025.s001.docx]

**Reviewer #1:** This paper compares the osteoporosis predictive ability of 2D and 3D assessments performed on CT scans. The prediction accuracy was assessed through comparisons between HUHA and T-score / BMD values extracted from DXA.

Some concerns arises throughout the manuscript, which in this Reviewer opinion should be deeply addressed by the authors.
Specific comments:

1. In the Introduction section, Authors state that the study was motivated by the “need to overcome the limitations and underusage of DXA”. Regarding underusage, a specific contextual framework should be added, as DXA is the gold standard and most popular method for osteoporosis screening in many countries around the world. Regarding limitations, these should be specified, especially considering that the evaluation of the accuracy of the proposed method is in fact exclusively based on the DXA-classification between healthy and osteoporotic patients. The predictive performance of the T-score has indeed demonstrated to be moderate and it is reported in literature that approximately half of the people suffering from a fracture presents non-osteoporotic T-score levels (/10.1007/s11914-011-0093-9). In this Reviewer opinion, this could definitely affect the present study and probably, different classification parameters, such as the femoral strength (/10.1016/j.compbiomed.2020.104093), could be more reliable.

🡪 Thank you for your kind advice. We added a specific contextual framework regarding underusage and limitations of DXA on the manuscript in the Introduction section by citing the references you recommend as follows.

[Response] “Despite dual-energy X-ray absorptiometry (DXA) is the gold-standard methods and the most commonly used technique to measure BMD, there are underusage of DXA due to lack of knowledge regarding risk. Because osteoporosis is asymptomatic until the patients sustain major incidental fragile fractures such as vertebral body or hip fractures. Moreover, patients at risk of the condition may not recognize its seriousness and avoid participating in screening programs voluntarily. In addition, there are several limitations of DXA screening associated with BMD measurements and diagnostic performance. BMD values can be affected by not only artifactually increased the BMD values due to degenerative disc disease, compression fracture, or aortic calcification, but also improper patient positioning or scan analysis. Furthermore, the predictive performance of the T-score demonstrated to be moderate in postmenopausal women, with approximately half of the people suffering from a fracture presents non-osteoporotic T-score levels [1].”

2. The rationale behind a two-dimensional analysis carried out on three-dimensional images is not clear and should be deepened: having CT available for the osteoporosis diagnosis is in fact rare, since DXA is the technique of choice in most cases. Why reduce the amount of information available by analyzing a single plane of the entire volume?

🡪 Thank you for your kind advice. We supposed to analyze both two-dimensional (2D) and three-dimensional (3D) images for diagnosing osteoporosis on APCT. Therefore, we analyzed 2D images with commercial three-dimensional analysis software to save the trouble of measuring twice in 2D ROI measurement on picture archiving and communication system (PACS) and 3D VOI measurement on analysis software. In proximal femur, 3D VOI measurement would bring better results because it has a complex structure known as the principal compressive and tensile groups, secondary compressive and tensile groups, greater trochanteric group, and Ward's triangle. However, as in this paper, special software is used to measure the entire volume of 3D proximal femur, and in contrast, 2D ROI measurement can be directly applied on PACS with more convenient access under daily practice and can be efficient screening method. In our study, there was no superiority between AUCs in 2D ROI and 3D VOI measurements (*P* > 0.05) and 2D ROI showed moderate to good agreement (*ICC* range: 0.63~0.84). Therefore, 2D ROI measurement can be used instead of 3D VOI measurements. We have added this issue in the Discussion section.

[Response]

In case where 3D VOI measurements are difficult, 2D ROI measurements can be performed instead in that it is more accessible and efficient screening method in daily practice by analyzing on picture archiving and communication system (PACS).

**Reviewer #2:**

1. Osteoporosis is derived from Greek, which literally means a bone with holes. It is defined by the World Health Organization (WHO) as “a systemic skeletal disease characterized by low bone mass and microarchitectural deterioration of bone tissue with a consequent increase in bone fragility and susceptibility to fracture”. Osteoporosis is not related with the fat, but with the bone or its absence (holes). it is confusing that you use HU at fat to predict osteoporosis. It makes more sense to use porosity of the bone or something similar.
**🡪** We appreciate your great comment. I respect your opinion. Although it has been understood for many years that marrow adiposity increases with age, this has historically been viewed as a neutral process, with the adipose tissue serving as a space filler in the bone marrow. However, recent studies indicate that marrow fat accumulation is part of dynamic processes that also affect bone density [2]. A shift in stem cell lineage allocation toward adipogenesis and away from osteoblastogenesis may contribute to age-related bone loss [3]. Consistent with these observations, clinical studies using different methods to assess marrow fat have found a negative correlation with bone density [4]. Previous studies have also indicated that an increase in the fat content of bone marrow was related to aging, osteoporosis, and menopause status in women [5]. In addition, previous studies have reported an association between prevalent vertebral fracture and higher vertebral marrow fat content measured by biopsy [6] and with magnetic resonance spectroscopy (MRS) [2, 7]. As a result, osteoporosis is associated with an increased marrow fat mass due to a shift of differentiation of mesenchymal stem cells to adipocytes rather than osteoblasts.

We thought that CT is a good modality to measure the marrow fat quantitatively using HU. HU histogram analysis (HUHA) enables the calculation of fat composition and their quantitative analysis with CT, similar to MRS. We demonstrated in previous studies that HUHA_fat_ is an important index to determine osteoporosis [8, 9]. Therefore, in this study, we want to compare the osteoporosis-predicting ability of computed tomography (CT) indexes in abdominal-pelvic CT.

2. Do your data include any patient with osteoporosis-related femur fracture?
**🡪** Thank you for your kind comment. There is no patient of osteoporosis-related femur fracture in our study.

3. Do the 3D-Femur-VOI and 2D coronal-ROI measurements are obtained manually? How much time did you need to do the measurements? Is it needed any special training to perform the analysis?

🡪 Thank you for your kind advice. 3D-Femur-VOI and 2D coronal-ROI measurements were performed manually without any special training on commercial three-dimensional analysis software. 3D VOI measurement took less than 5 minutes per patient and 2D ROI measurement took less than 1 minute.

4. You evaluate the 3D-Femur-VOI for the total femur but the 2D coronal-ROI only for the femoral neck. Have you study other 2D regions?
🡪 Thank you for your kind advice. The diagnosis of osteoporosis is based on the T-score of the lumbar spine or femoral neck. Since the BMD value of the femur neck was used as a reference in our study and which was obtained from the same image plane with 2D coronal-ROI on APCT, 2D-coronal-ROI values were obtained from the femoral neck and not measured in other regions of the femur.

5. It is not clear the figure's caption. They are repeated. It will be easier if you divide the captions.
🡪 Thank you for your kind remarks. I divided captions as you suggested.

6. One of the limitations of standard analysis of DXA images is that they do not differentiate between trabecular and cortical bone. This could be solved with CT images. Do you analyze differences between cortical and trabecular bone?

🡪 Thank you for your comments. In fact, we can measure Hounsfield unit (HU) of the cortical and trabecular bone separately on CT scan. Schwartz et al. reported higher marrow fat correlated with lower trabecular, but not cortical by quantitative computed tomography [2]. However, analyzing cortical and trabecular bone separately is not implemented in this paper because it is a time-consuming task and may become a study that is far from the purpose of our study. Furthermore, unlike determining the threshold value of the fat component (0 HU or less), the threshold of the trabecular bone component was difficult to determine. In our study, HU histogram analysis (HUHA) enables the calculation of the compositions of fat and bone components according to HU spectrum, however, it is not easy to discriminate cortical and trabecular bone from entire bone based on arbitrarily setting a threshold value of HU. As you commented, we have further explained on Limitation section as follows.

[Response]

Fourth, we did not analyze the cortical and trabecular bone separately in our study. Because it is a time-consuming task and may become a study that is far from the purpose of our study. Furthermore, unlike determining the threshold value of the fat component (0 HU or less), the threshold of the trabecular bone component was difficult to determine.

**REFERENCES**

1.	Baim S, Leslie WD. Assessment of fracture risk. Current osteoporosis reports. 2012;10(1):28-41.

2.	Schwartz AV, Sigurdsson S, Hue TF, Lang TF, Harris TB, Rosen CJ, et al. Vertebral bone marrow fat associated with lower trabecular BMD and prevalent vertebral fracture in older adults. The Journal of Clinical Endocrinology & Metabolism. 2013;98(6):2294-300.

3.	Moerman EJ, Teng K, Lipschitz DA, Lecka‐Czernik B. Aging activates adipogenic and suppresses osteogenic programs in mesenchymal marrow stroma/stem cells: the role of PPAR‐γ2 transcription factor and TGF‐β/BMP signaling pathways. Aging cell. 2004;3(6):379-89.

4.	Sheu Y, Cauley JA. The role of bone marrow and visceral fat on bone metabolism. Current osteoporosis reports. 2011;9(2):67-75.

5.	Tang G, Lv Z, Tang R, Liu Y, Peng Y, Li W, et al. Evaluation of MR spectroscopy and diffusion-weighted MRI in detecting bone marrow changes in postmenopausal women with osteoporosis. Clinical radiology. 2010;65(5):377-81.

6.	Justesen J, Stenderup K, Ebbesen E, Mosekilde L, Steiniche T, Kassem M. Adipocyte tissue volume in bone marrow is increased with aging and in patients with osteoporosis. Biogerontology. 2001;2(3):165-71.

7.	Wehrli FW, Hopkins JA, Hwang SN, Song HK, Snyder PJ, Haddad JG. Cross-sectional study of osteopenia with quantitative MR imaging and bone densitometry. Radiology. 2000;217(2):527-38.

8.	Lim HK, Ha HI, Park S-Y, Lee K. Comparison of the diagnostic performance of CT Hounsfield unit histogram analysis and dual-energy X-ray absorptiometry in predicting osteoporosis of the femur. European radiology. 2019;29(4):1831-40.

9.	Lee H-W, Ha HI, Park S-Y, Lim HK. Reliability of 3D image analysis and influence of contrast medium administration on measurement of Hounsfield unit values of the proximal femur. Plos one. 2020;15(10):e0241012.
