# Supplementary material for: Comparison of diagnostic accuracy of 2D and 3D measurements to determine opportunistic screening of osteoporosis using the proximal femur on abdomen-pelvic CT
Source: PLoS One. 2022 Jan 4;17(1):e0262025. doi: 10.1371/journal.pone.0262025 (PMC8726491; doi:10.1371/journal.pone.0262025)
Supplement: Attachment — Submitted filename: Reviewer comments-minor review.docx [file pone.0262025.s002.docx]

**Reviewer #1**: The Authors responded satisfactorily and precisely to each comment and the requested additional information have been added in the manuscript. The result is a significantly improved manuscript.

- Thank you for your consideration.

**Reviewer #2**: The authors have done a great work and answer the most of the questions properly, however, some concerns remained:
1. In my opinion CT scans have several advantages against DXA scans to osteoporosis diagnosis and monitoring, but they are not properly described on the paper. The World Health Organization (WHO) has established DXA as the best densitometric technique for assessing BMD in postmenopausal women and based the definitions of osteopenia and osteoporosis on its results. In fact, you use DXA T-score to evaluate your measurements. So, I wouldn’t say that one of the limitations of DXA is its underusage for osteoporosis diagnosis. CT scans radiation is higher, and they are more expensive than DXA scans; therefore, I don’t see the fact that CT scans could substitute DXA for osteoporosis screening. Other screening tools as ultrasound or DXA-based 3D modelling techniques could be a better alternative. Include these methods in the state of the art. This doesn’t mean opportunistic screening of osteoporosis using CT couldn’t be beneficial. Main advantages of CT are the 3D measurements and the possibility to evaluate bone compartments separately, and those must be addressed on the paper.

**🡪 Thank you for your kind advice. As you mentioned, I agree with your opinion that DXA is a gold standard, and our study also use DXA T-score as a reference standard because it is a retrospective study and DXA is the only recognized standard for diagnosing osteoporosis. The word “underusage” in Introduction section has already been used in phrases from previous studies regarding DXA screening in our references [1-3]. They reported that DXA screening was underused in women at increased fracture risk, including women aged ≥ 65 years. Meanwhile, DXA screening was common among women at low fracture risk, such as younger women without osteoporosis risk factors [1]. The inefficiency of osteoporosis screening using DXA is well known through several studies, and among women over 65 years who underwent APCT in our institution, there are not many patients who underwent DXA. Therefore, we agree with previous opinion that alternative techniques are needed to increase osteoporosis detection, and this is our intention. So, in order to better convey our meaning, we have modified the 2^nd^ paragraph of Introduction section and word “underusage” was deleted.**

**Next, in our study, the concept of “opportunistic” screening of APCT is to overcome the low participation in screening for osteoporosis with DXA, not to replace DXA. APCT is one of the most commonly and widely performed imaging in adults for identification of routine health checkups, various diseases, or follow-up assessments. For patients undergoing APCT, opportunistic osteoporosis screening has been proposed for concurrent BMD assessment by measuring Hounsfield units (HU) in lumbar spine or femur neck without any additional imaging, radiation exposure, or appointments. In the process of analyzing HU on APCT, we want to find out the measurement method and reliability of measurements obtained with 2D-ROI and 3D-VOI on APCT at femur neck. Therefore, this is the purpose of our study, and this paper was not written for the usefulness of screening for osteoporosis using APCT.**

**Finally, we agree your opinion that the biggest advantage of CT scan is 3D measurement and possibility to evaluate bone component, since it is an important matter related to our study, we added this sentence in 3^rd^ paragraph of Introduction section.**

[Response] 2^nd^ and 3^rd^ paragraph of introduction section

Dual-energy X-ray absorptiometry (DXA) is recognized as the reference method to measure bone mineral density (BMD) for osteoporosis diagnosis. The World Health Organization (WHO) has established DXA as the best densitometric technique for assessing BMD at the hip and lumbar spine for osteoporosis screening. Unfortunately, several studies have shown that DXA screening are performed less frequently in high-risk populations including women aged ≥ 65 years, and more commonly in women at low fracture risk without osteoporosis risk [1, 2, 4]. In addition, DXA is a two-dimensional (2D) technique, clinically relevant diagnostic errors can be made: the presence of degenerative disc disease, compression fracture, or aortic calcification may increase the bone density without improving the actual skeletal strength and can be sources of errors in the diagnosis of osteoporosis. Therefore, there is a growing appreciation of the need for alternative screening methods.

Several studies have yielded optimistic results using Abdomen-pelvic CT (APCT) for opportunistic screening of osteoporosis. APCT is one of the most commonly and widely performed imaging in adults for identification of routine health checkups, various diseases, or follow-up assessments. Using APCT, BMD can be assessed in lumbar spine or femur by measuring Hounsfield units (HU) without need for any additional imaging, radiation exposure, or patient time. Even if a small number of these scans were used for opportunistic screening of osteoporosis, the impact could be substantial. In addition, APCT evaluate bone component separately and discriminate bone microarchitecture with high resolution.

2. As fracture is one of the main outcomes of osteoporosis, you should specify that your data do not include any osteoporosis fracture, how are you explore that and why patients with fracture are not included in the study.

**🡪 Thank you for your kind advice. Because we collected patients who underwent APCT, there were not many patients who had acute osteoporotic fracture at the time of APCT, and there were 5 cases who underwent APCT after surgery including total hip arthroplasty or internal nailing. However, five of these patients were excluded from this study because HU couldn’t be measured due to metallic artifacts. In addition, among the patients mentioned in this paper, 27 patients underwent APCT for slip-down and fall-down injury, and some of these patients had osteoporotic fractures. In these cases, HU was measured in the unbroken contralateral femur.**

**Also, as mentioned in #1, it has nothing to do with the purpose of this study. I agree with your opinion that osteoporotic fracture of proximal femur is one of the main outcomes of osteoporosis. Therefore, we researched it and our studies about HU histogram analysis and BMD for proximal femoral fragility fracture have been published to European radiology recently. If you are interested, please refer to it [5].**

3. On Materials and Methods: “Osteoporosis was defined as a T-score ≤ −2.5 and nonosteoporosis was defined as a T-score > −2.5 [18].”
a. I don’t think this reference suits better for this definition. You should use WHO or IOF references.

**🡪 Thank you for your kind advice. We change the reference and sentences as you mentioned as follows.**

[Response]

T-score was interpreted as osteoporosis (*T*-score ≤ −2.5), osteopenia ( −2.5*< T*-score < −1.0), and normal (*T*-score $\geq$ −1.0) [6]. Patients were regrouped into osteoporosis (*T*-score ≤ −2.5) and non-osteoporosis groups (*T*-score > −2.5).

b. What about low bone density or osteopenia patients? Have you tested? Those are the more interested to detect for a clinical point of view. Besides they are more difficult to diagnose and monitor using only DXA scans.

**🡪 We deeply appreciate your great comments. However, we have not tested low bone density or osteopenia patients in our study. Dual-energy x-ray absorptiometry (DXA) is a technique used to aid in the diagnosis of osteopenia and osteoporosis. However, the purpose of our study is to find osteoporosis patients by analyzing APCT previously performed for various medical cause, unlike independently performing DXA for osteoporosis screening. We thought that APCT has a potential opportunity for concurrent BMD screening of the lumbar spine or femur without the need for any additional imaging, radiation exposure, or patient time. Further, in our country, osteopenia is not covered by insurance. Therefore, we focus on detect osteoporosis patients on APCT as an opportunistic screening and evaluate the reliability of measurements in two- and three-dimensional analyses on APCT.**

**Osteoporosis is asymptomatic until patients undergo major accidental fragility fractures such as vertebral or hip fractures. Therefore, if osteopenia is suspected on CT scan, it will be helpful for the patient’s treatment plan and prognosis if the clinician is informed.**

4. Age is highly related with osteoporosis. In fact, you have statistical significances between osteoporotic and nonosteoporotic patients (Table I). Have you tested your statistics in a age-matched cohort? Or removing age effect? If not, you should indicate in the discussion.

**🡪 Thank you for pointing this out. In fact, as you mentioned, we thought to conduct the study with an age-matched cohort, but it was not easy to collect a control group because age is a factor highly related to osteoporosis. In other words, it was not easy to find age-matched patients who were actually old-aged and did not have osteoporosis. So, the study was conducted by collecting consecutive patients, and there was no choice but to make a statistical difference between osteoporosis and non-osteoporotic patients. Therefore, we mentioned this limitation to the Discussion section.**

[Response]

Fifth, this study wasn’t really a case control but a cohort study involving two groups: osteoporosis and non-osteoporotic patients. Matching is one of the methods intended to eliminate confounding such as age. However, in this study, it is impossible to select age-matched cohort who were old-aged and did not have osteoporosis. Therefore, consecutive patients were selected for within group comparisons.

5. In the discussion you write: “The primary goal of our study was to determine whether the diagnostic performance for evaluation of osteoporosis differs depending on the 2D or 3D measurement.” Do you refer to both measurements on CT or 2D-DXA vs. 3D-CT? You should clarify.

**🡪 Thank you for your kind advice. We added the word “APCT” and change the sentence as follows.**

[Response]

The primary goal of our study was to determine whether the diagnostic performance for evaluation of osteoporosis differs depending on the 2D or 3D measurement on APCT.”

6. Differences between your results and other studies on the literature are not clear. Degenerative changes and aortic veins at the lumbar spine may lead an overestimation of the BMD measured at the DXA but not affect to QCT measurements. Racial differences shouldn’t affect to the accuracy of a technique.

**🡪 Thank you for your kind advice. Since it is an unclear paragraph, we edited this paragraph by adding a few sentences and changing some words. Studies on opportunistic screening of osteoporosis using APCT have recently been published, however, showed different results regarding diagnostic performance and threshold HU. Therefore, in this paragraph, we tried to explain the reason why the results were different among the studies using APCT. First, the measurement locations were different. In our study, we measured the ROI on the femur, while other papers studied the lumbar spine. Second, racial differences may contribute to this difference of results. Our study cohort consisted of only Asian women, and Asians have been reported to have lower BMDs in comparison with Africans, Hispanics, and Caucasians [7]. In fact, BMD and T-score are recommended to be adjusted according to each region and race [7]. Similarly, threshold CT HU are affected by gender and race, and diagnostic performance can be changed. Therefore, the influence of gender and race must be considered when interpreting CT HU values.**

[Response]

Studies on opportunistic screening of osteoporosis using APCT have recently been published, however, showed different results regarding diagnostic performance and threshold HU. Pickhardt et al. reported the diagnostic performance of the mean CT HU value for diagnosing osteoporosis with an AUC of 0.83, sensitivity of 76%, and specificity of 75% at a 135-HU threshold for the lumbar spine in American population. Alacreau et al. reported an AUC of 0.66, sensitivity of 91.4%, and specificity of 58% at a 160-HU threshold for the L1 body in Southern European population. In the present study, mean-CT HU of 3D-VOI measurement showed an AUC of 0.96, sensitivity of 94.8%, specificity of 85% at a 231-HU threshold, and mean-CT HU of 2D-ROI measurement showed an AUC of 0.95, sensitivity of 89.6%, specificity of 87.4% at a 180-HU threshold at femoral neck. These differences may be due to the following reasons. First, the measurement locations were different. We used the femur instead of the lumbar spine. In fact, the distribution of cancellous bone is different in the lumbar and femur neck. In previous study, lower BMD for lumbar spine was more prevalent [8]. Another explanation is that weight-bearing can raise in bone density especially in the femur region [8, 9]. Second, racial differences may contribute to this difference of results. Our study cohort consisted of only Asian women, and Asians have been reported to have lower BMDs in comparison with Africans, Hispanics, and Caucasians. Similarly, ethnic differences might contribute to the discrepancy of CT attenuation thresholds in each study.

References

1.	Pickhardt, P.J., et al., *Simultaneous screening for osteoporosis at CT colonography: bone mineral density assessment using MDCT attenuation techniques compared with the DXA reference standard.* Journal of Bone and Mineral Research, 2011. **26**(9): p. 2194-2203.

2.	Amarnath, A.L.D., et al., *Underuse and overuse of osteoporosis screening in a regional health system: a retrospective cohort study.* Journal of general internal medicine, 2015. **30**(12): p. 1733-1740.

3.	Elliot-Gibson, V., et al., *Practice patterns in the diagnosis and treatment of osteoporosis after a fragility fracture: a systematic review.* Osteoporosis international, 2004. **15**(10): p. 767-778.

4.	Curtis, J.R., et al., *Longitudinal trends in use of bone mass measurement among older Americans, 1999–2005.* Journal of Bone and Mineral Research, 2008. **23**(7): p. 1061-1067.

5.	Park, S.-Y., et al., *Comparison of HU histogram analysis and BMD for proximal femoral fragility fracture assessment: a retrospective single-center case–control study.* European Radiology, 2021: p. 1-8.

6.	Lewiecki, E.M., et al., *International Society for Clinical Densitometry 2007 Adult and Pediatric Official Positions.* Bone, 2008. **43**(6): p. 1115-21.

7.	Cauley, J.A., *Defining ethnic and racial differences in osteoporosis and fragility fractures.* Clinical Orthopaedics and Related Research®, 2011. **469**(7): p. 1891-1899.

8.	Mounach, A., et al. *Discordance between hip and spine bone mineral density measurement using DXA: prevalence and risk factors*. in *Seminars in arthritis and rheumatism*. 2009. Elsevier.

9.	Kohrt, W.M., et al., *Additive effects of weight‐bearing exercise and estrogen on bone mineral density in older women.* Journal of Bone and Mineral Research, 1995. **10**(9): p. 1303-1311.
